# Phenolic Compound Ethyl 3,4-Dihydroxybenzoate Retards Drug Efflux and Potentiates Antibiotic Activity

**DOI:** 10.3390/antibiotics11040497

**Published:** 2022-04-08

**Authors:** Wen-Jung Lu, Yan-Jyun Huang, Hsuan-Ju Lin, Chun-Ju Chang, Pang-Hung Hsu, Gui-Xia Ooi, Mei-Ying Huang, Hong-Ting Victor Lin

**Affiliations:** 1 Department of Food Science, National Taiwan Ocean University, No. 2, Pei-Ning Road, Keelung 202, Taiwan; miss350100@gmail.com (W.-J.L.); zxc214863@gmail.com (Y.-J.H.); angel810801@gmail.com (H.-J.L.); chunju@mail.ntou.edu.tw (C.-J.C.); guixia95@gmail.com (G.-X.O.); 2 Center of Excellence for the Oceans, National Taiwan Ocean University, No. 2, Pei-Ning Road, Keelung 202, Taiwan; phsu@mail.ntou.edu.tw; 3 Department of Bioscience and Biotechnology, National Taiwan Ocean University, No. 2, Pei-Ning Road, Keelung 202, Taiwan; 4 Institute of Biochemistry and Molecular Biology, National Yang Ming Chiao Tung University, No. 155, Linong Street, Taipei 112, Taiwan; 5 Division of Aquaculture, Fisheries Research Institute, Council of Agriculture, No. 199, Hou-Ih Road, Keelung 202, Taiwan; myhuang@mail.tfrin.gov.tw

**Keywords:** ethyl 3,4-dihydroxybenzoate, efflux pump inhibitors, multidrug resistance, drug transporters, phenolic compounds, molecular docking

## Abstract

The World Health Organization indicated that antibiotic resistance is one of the greatest threats to health, food security, and development in the world. Drug resistance efflux pumps are essential for antibiotic resistance in bacteria. Here, we evaluated the plant phenolic compound ethyl 3,4-dihydroxybenzoate (EDHB) for its efflux pump inhibitory (EPI) activity against drug-resistant *Escherichia coli*. The half-maximal inhibitory concentration, modulation assays, and time-kill studies indicated that EDHB has limited antibacterial activity but can potentiate the activity of antibiotics for drug-resistant *E. coli*. Dye accumulation/efflux and MALDI-TOF studies showed that EDHB not only significantly increases dye accumulation and reduces dye efflux but also increases the extracellular amount of antibiotics in the drug-resistant *E. coli*, indicating its interference with substrate translocation via a bacterial efflux pump. Molecular docking analysis using AutoDock Vina indicated that EDHB putatively posed within the distal binding pocket of AcrB and in close interaction with the residues by H-bonds and hydrophobic contacts. Additionally, EDHB showed an elevated postantibiotic effect on drug-resistant *E. coli*. Our toxicity assays showed that EDHB did not change the bacterial membrane permeability and exhibited mild human cell toxicity. In summary, these findings indicate that EDHB could serve as a potential EPI for drug-resistant *E. coli*.

## 1. Introduction

The World Health Organization indicated that antibiotic resistance is one of the greatest threats to health, food security, and development worldwide. A growing number of bacterial infections are becoming harder to treat owing to antibiotic resistance. Drug efflux pumps are one of the major elements contributing to the antibiotic resistance of bacteria. The efflux of antibiotics via the bacterial cell wall into extracellular spaces by the membrane-bound drug efflux pump could reduce drug accumulation in the bacteria, increasing drug resistance and survival rate of the bacteria [1]. For example, the Major Facilitator Superfamily transporter NorA could confer resistance against fluoroquinolones and biocides in the Gram-positive bacteria *Staphylococcus aureus* [2], and the AcrAB-TolC tripartite transporter could confer multidrug resistance in the Gram-negative bacteria *Escherichia coli* by excreting a wide variety of antibiotics, including fluoroquinolones, and macrolides [3].

The Interagency Coordination Group on Antimicrobial Resistance [4] indicated that 10 million people could die each year as a result of drug-resistant diseases by 2050 if no action is taken. However, costly and risky preclinical stages of new antibiotic research have limited the success of the release of new antibiotics from pharmaceutical companies [5]. In recent years, the use of efflux pump inhibitors (EPIs) gained increasing attention as a promising approach for the treatment of infections caused by pathogens expressing drug efflux pumps [6]. They have been considered as a potential therapeutic agent to be used in adjunctive therapy that can restore the activity of antibiotics that are no longer effective against pathogens, via interfering with the bacterial efflux pumps during treatment. The modes of actions for EPIs to increase the antibiotic activity are (i) to serve as a competitive/noncompetitive inhibitor for drug efflux, (ii) to interfere with efflux pump expression or assembly, and (iii) to interrupt the energy source for active drug efflux [7]. Well-known EPIs, such as proton uncoupler carbonyl cyanide *m*-chlorophenylhydrazone (CCCP) and competitive inhibitor phenylalanine–arginine β-naphthylamide (PAβN), exhibit synergistic effects with various antibiotics, though their cellular toxicity might have limited their uses in clinical treatments [6]. Consequently, scientists have been screening for potential EPIs of plant, algal, and microbial origins [6,8]. Several classes of EPIs, including alkaloids [9], amide derivatives [10,11], antibiotic analogs [12,13], flavonoids [14,15], and terpenes [16], were reported. Still, very few natural EPIs for Gram-negative bacteria have been identified [6,17,18].

Phenolic compound ethyl 3,4-dihydroxybenzoate (EDHB) was reported to be present in plants and foods, such as peanut and wine [19]. EDHB was reported to possess antibacterial, antioxidant, and anticancer activities [20]. To our knowledge, no reports have been found to evaluate its potential as an EPI. In this study, we evaluated its antibiotic potentiating activity by using modulation assay and time-kill assays. Additionally, we determined its EPI activity via dye accumulation assays, dye efflux assays, and MALDI-TOF analysis. Furthermore, we investigated the impact of EDHB on the cell membrane by a membrane permeability assay and determined its postantibiotic effect (PAE). Finally, we evaluated its possible cytotoxicity against human cell lines.

## 2. Results and Discussion

### 2.1. Half-Maximal Inhibitory Concentration (IC_50_) and Modulation Assay for EDHB against Drug-Resistant E. coli

In a broth dilution assay, when bacteria overexpress an efflux transporter, they are less susceptible to tested chemical compounds, indicating the efflux activity of the transporter [21]. *E. coli* efflux pump AcrB has been reported to confer resistance against antibiotic ciprofloxacin, clarithromycin, and erythromycin [22,23]. In this study, the construct *E. coli* Kam3 and Kam3 harboring pSYC-*acrB* (Kam3-AcrB) was used in the IC_50_ and modulation assays.

The IC_50_ of EDHB for Kam3 and Kam3-AcrB was determined to be 500 µg/mL (data not shown), indicating its mild antibacterial activity. Table 1 shows that the IC_50_ of clarithromycin, erythromycin, and ciprofloxacin was determined to be 175, 125, and 0.06 µg/mL, respectively. Our modulation assay indicated that RND transporter inhibitor PAβN at 20 µg/mL could reduce the IC_50_ of erythromycin and clarithromycin by eightfold and fourfold. EDHB at 125 µg/mL could reduce the IC_50_ of clarithromycin by fourfold. Similarly, EDHB at 31.25 µg/mL could reduce the IC_50_ of erythromycin by fourfold. EDHB at 3.9 µg/mL could reduce the IC_50_ of ciprofloxacin by twofold. Intriguingly, for the Kam3 cells (*acrB* deletion) not expressing AcrB, EDHB could reduce the IC_50_ of clarithromycin by twofold; in addition, EDHB did not reduce the IC_50_ of erythromycin and ciprofloxacin. Our modulation results show that EDHB has higher modulation factors of the antibiotics in Kam3-AcrB than those in Kam3. Overall, our IC_50_ and modulation results suggest that EDHB could modulate the activities of clarithromycin, erythromycin, and fluoroquinolone against Kam3-AcrB.

Plants are natural and sustainable, and their bioactive substances are still largely unexplored [24], making them a promising source of novel EPIs. Alkaloid piperine from the peppers (*Piper nigrum* and *Piper longum*) has been reported to potentiate the activity of ciprofloxacin against the *S. aureus* strains expressing drug efflux pumps [9]. The pungent chemical compound capsaicin (8-methyl-N-vanillyl-6-nonenamide) of hot chilies (genus *Capsicum*) was shown to significantly reduce the minimum inhibitory concentration of ciprofloxacin by interfering with the NorA transporter of *S. aureus* [25].

### 2.2. Effect of EDHB on Time-Kill Curves

**Figure 1:**
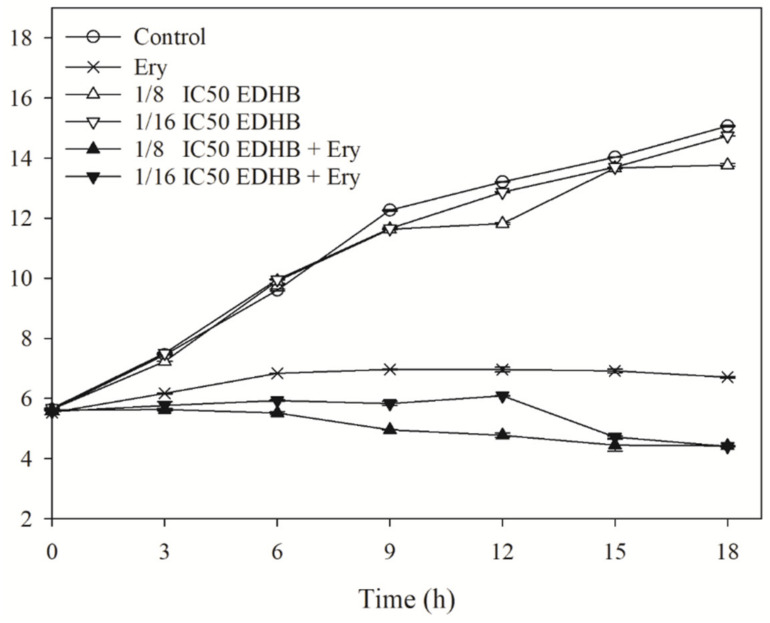
Time-kill curve of *E. coli* Kam3-AcrB with Ery, EDHB, and in combination. Ery, Erythromycin. Data are expressed as mean ± SD (*n* = 3).

To monitor the growth of Kam-AcrB in the presence of erythromycin and erythromycin + EDHB, time-kill studies were used to observe the changes in the actual cell counts of the bacteria after exposure to erythromycin and erythromycin + EDHB during a period of time. Figure 1 shows that the *E. coli* Kam3-AcrB (control) along with the Kam3-AcrB incubated with 1/8 IC_50_ EDHB or 1/16 IC_50_ EDHB showed similar cell growth after 18 h. The results indicate that the sub-IC_50_ concentration of EDHB did not reduce the growth of Kam3-AcrB, which was consistent with our IC_50_ data.

Erythromycin is a bacteriostatic antibiotic, and the addition of erythromycin to the Kam3-AcrB culture significantly inhibited the growth of *E. coli* Kam3-AcrB, with a cell count of 6.71 ± 0.03 Log CFU/mL at 18 h. Intriguingly, the combined use of erythromycin and EDHB at a concentration of its 1/8 and 1/16 IC_50_ exhibited a better inhibitory activity for the growth of *E. coli* Kam3-AcrB, with a cell count of Log 4.42 ± 0.01 and Log 4.40 ± 0.01 CFU/mL, respectively, at 18 h. A decrease of cell counts was observed with the addition of 1/8 and 1/16 IC_50_ EDHB after 6 h and 12 h, respectively, indicating its potentiation activity. This was consistent with our modulation assay results in which the addition of EDHB exhibited a synergistic effect on the application of erythromycin. In the 18 h cultivation, regrowth of the *E. coli* Kam3-AcrB was not observed in the presence of both erythromycin and erythromycin + EDHB.

### 2.3. Fluorescent Dye Accumulation Reduced by EDHB

**Figure 2:**
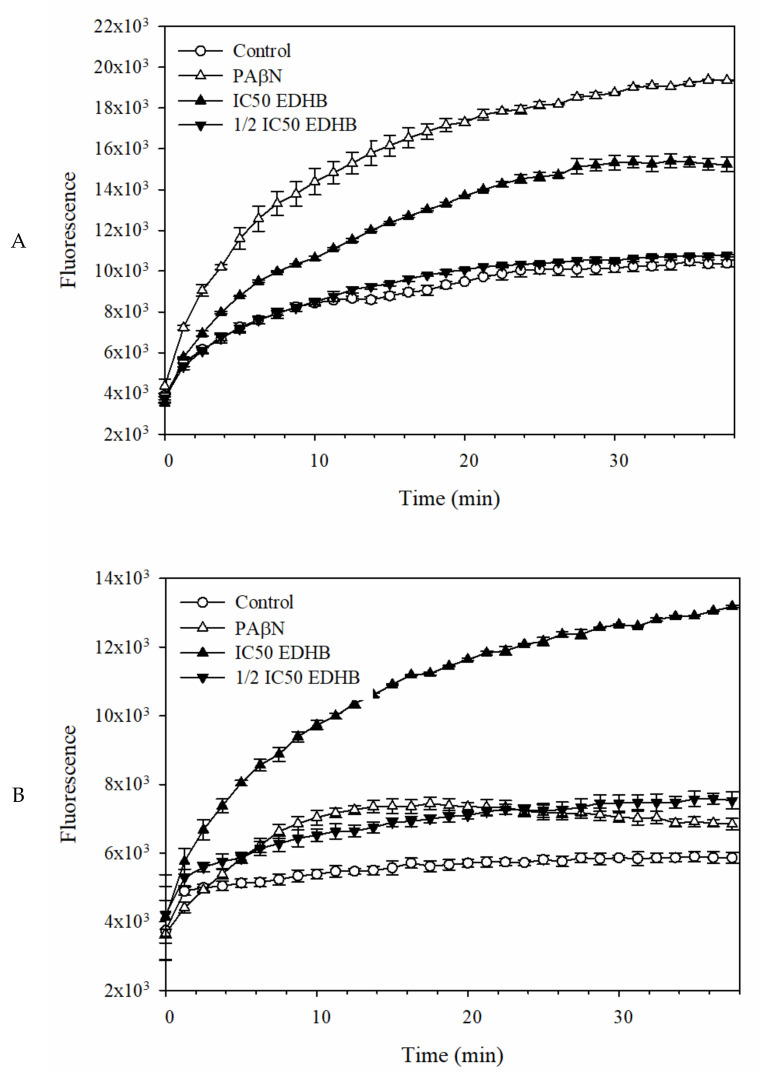
(**A**) H33342 and (**B**) EtBr accumulation of EDHB in *E. coli* Kam3-AcrB. The IC_50_ of EDHB is 500 µg/mL. Data are expressed as mean ± SD (*n* = 3).

Fluorescent dye accumulation assay was frequently used to evaluate amounts of substrates accumulated in the cells, and these amounts imply the level of substrates efflux by the detected cells expressing drug transporters [26]. Dyes ethidium bromide (EB) [27] and Hoechst 33342 (H33342) [28] show stronger fluorescence when bound to DNA and/or in a hydrophobic environment. In this study, dyes H33342 and EB were used to monitor the substrate accumulation in *E. coli* Kam3-AcrB, and the Resistance-Nodulation-Division (RND) pump modulator PAβN was used as the positive control group [29]. Figure 2 shows that the addition of EPI PAβN increases the accumulation of H33342 and EB in the *E. coli* Kam3-AcrB compared with that in the control (no addition of EPIs). Similar patterns were observed with the addition of the putative EPI EDHB. At IC_50_ of EDHB could increase the accumulation level of H33342, in contrast, 1/2 IC_50_ of EDHB showed no effect on the efflux of H33342 (Figure 2A) At both IC_50_ and 1/2 IC_50_, EDHB could increase the fluorescence of EB (Figure 2B) in Kam3-AcrB in a dose-dependent manner, providing a piece of indirect evidence that EDHB could interfere with the efflux of EB by Kam3-AcrB. Potential EPI daidzein, a soybean isoflavonoid, was shown to potentiate the activities of levofloxacin and carbenicillin and increase the accumulation of dye EB in a drug-resistant *E. coli* strain [30]. Our dye accumulation data indicate that EDHB could serve as a potential EPI for *E. coli* Kam3-AcrB.

### 2.4. Dye Efflux by Kam3-AcrB was Reduced by EDHB

The efflux assay is performed to study the efflux capacity of transporters by incubating cells with preloaded dyes that diffuse into the cells and monitoring the change of dye fluorescence after the addition of glucose to energize the cells along with the efflux pumps [31], providing a real-time efflux curve of the dye mediated by transporters. To further evaluate the potential of EDHB as an EPI for *E. coli* Kam3-AcrB, we monitored the H33342 and EtBr efflux in Kam3-AcrB in the presence of EDHB.

**Figure 3:**
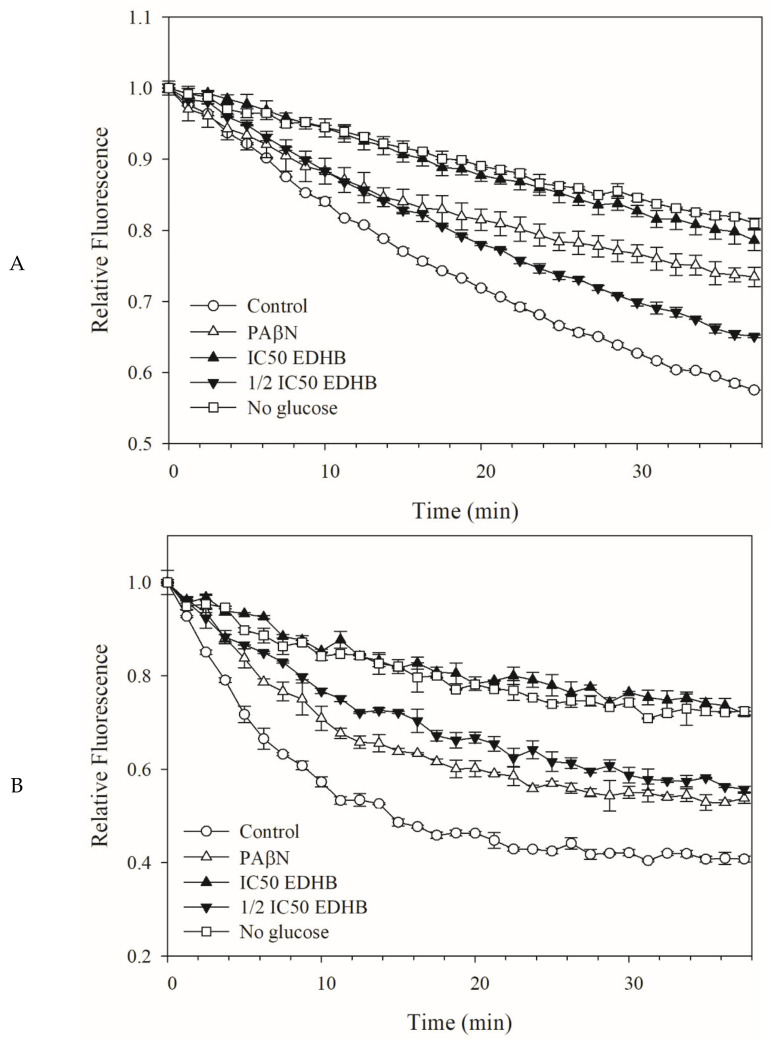
The (**A**) H33342 and (**B**) EtBr efflux assay with EDHB in *E. coli* Kam3-AcrB. The drug-resistant *E. coli* cells were added with glucose (25 mM) and H33342 (3 µM), EB (3 µM) in the presence or absence of PAβN (20 µg/mL) or EDHB (IC_50_ at 500 µg/mL). Data are expressed as mean ± SD (*n* = 3).

Figure 3A shows that the Kam3-AcrB (control) gradually reduced the H33342 fluorescence from time = 0 to reach a relative fluorescence of 0.57 at 38 min, indicating a continuous efflux of H33342 from the *E. coli* cells. The Kam3-AcrB without glucose energization reached an H33342 fluorescence of 0.84 at 38 min, which was higher than the control group, indicating that a limited energy source reduced the dye efflux. Additionally, the presence of pump inhibitor PAβN reduced the H33342 efflux, with an end relative fluorescence of 0.73 ± 0.01 at 38 min, indicating that PAβN could interfere with the efflux pump. The addition of EDHB (IC_50_ and 1/2 IC_50_) was shown to slow the decrease of H33342 fluorescence in a dose-dependent manner, as a result of its reduced H33342 efflux from the cells, possibly by interfering with the efflux pump AcrB. Similar results are observed in Figure 3B, in which the effect of EDHB on the efflux of EB was monitored. The addition of EDHB (IC_50_ and 1/2 IC_50_) also slowed the decrease of EB fluorescence, suggesting its interference with the EtBr efflux from the cells.

Plant-derived phenolic compounds comprising a single aromatic ring, such as capsaicin, cumin, gallic acid, olympicin A, and salicylic acid, showed interference with the efflux pumps in the Gram-positive bacterium *S. aureus*. For example, Kalia et al. [25] showed that the addition of capsaicin to *S. aureus* expressing NorA could significantly retard the fluorescence decrease of EtBr, suggesting a strong interference with EtBr efflux by capsaicin. However, very limited plant-derived phenolic compounds have been investigated for their EPI activity for Gram-negative bacteria. Our dye efflux results are consistent with our data in dye accumulation assays, indicating that EDHB could interfere with efflux of H33342 and EB, therefore suggesting its potential as an EPI for *E. coli* Kam3-AcrB.

### 2.5. Drug Efflux Interference by EDHB Monitored Using MALDI-TOF

**Figure 4:**
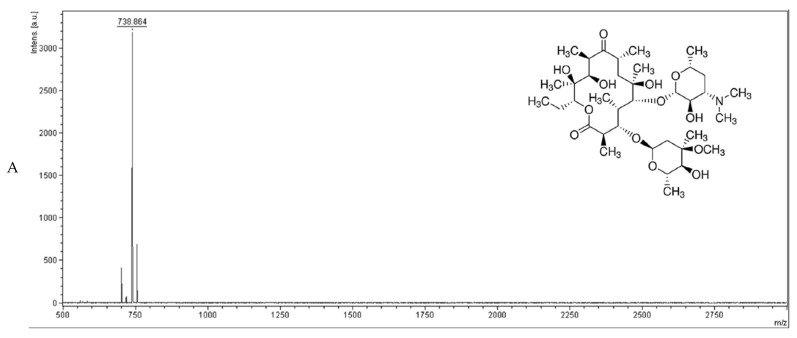

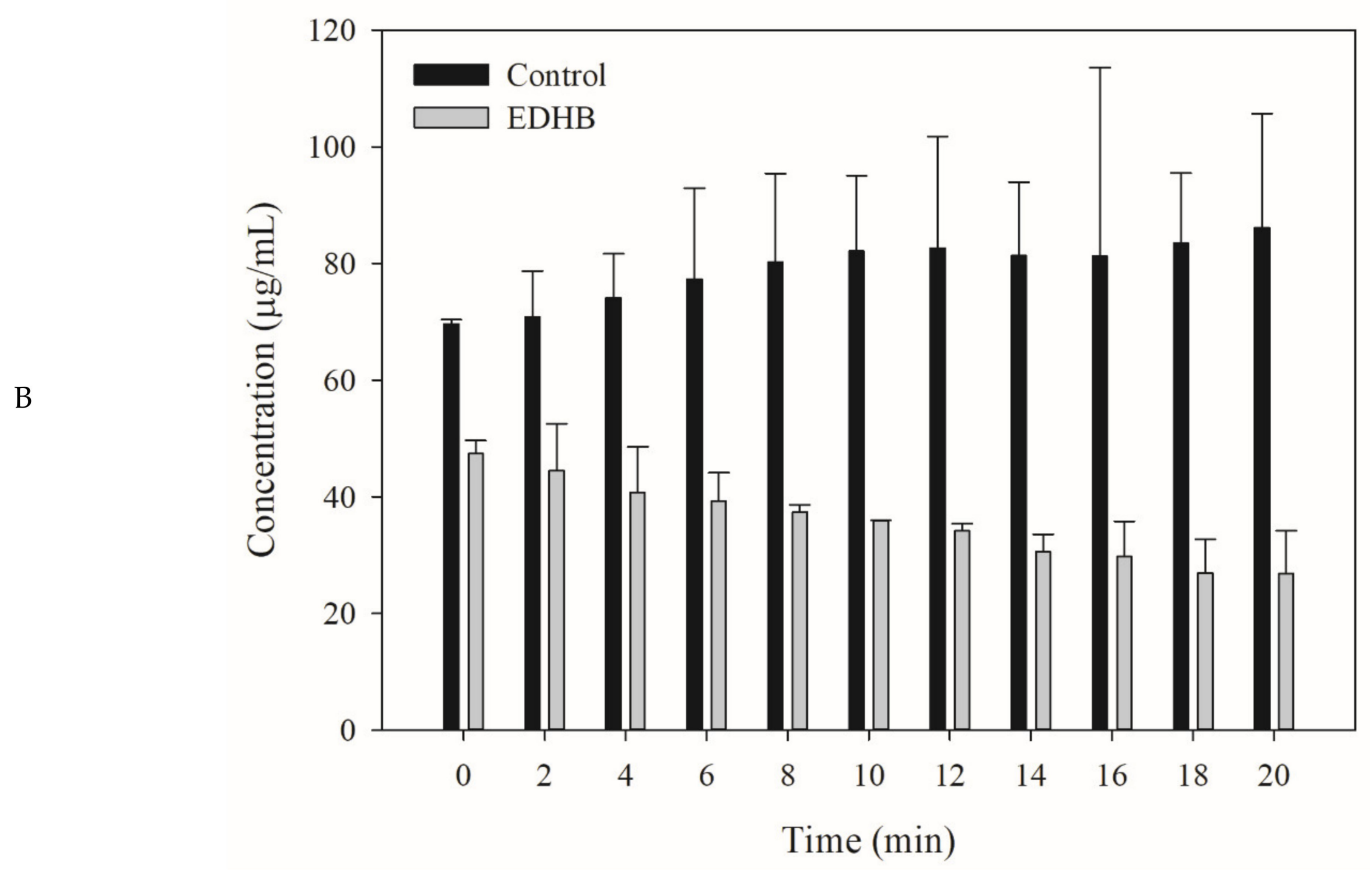
The erythromycin efflux activity of *E. coli* Kam3-AcrB was detected by using MALDI-TOF MS in the presence of EDHB. (**A**) The mass spectrum of erythromycin and (**B**) the intensity of extracellular erythromycin. The intensity was plotted at the main peak at m/z 738.35 of erythromycin, and the detection period was 20 min. Values are expressed as mean ± SD (*n* = 3).

Our modulation test indicated that EDHB could potentiate the activity of clarithromycin, erythromycin, and ciprofloxacin against *E. coli* Kam3-AcrB, and the dye accumulation/efflux data indicate that EDHB could interfere with dye efflux within the *E. coli* cells. However, these results do not necessarily mean that EDHB could interfere with antibiotic efflux in *E. coli*. Thus, MALDI-TOF MS was used to monitor the efflux of erythromycin in the extracellular space in the presence of EDHB. Figure 4A shows that the mass spectrum of erythromycin exhibits the main peak at *m*/*z* 738.864. The efflux of erythromycin by *E. coli* Kam-AcrB was measured by monitoring the concentration changes of extracellular erythromycin over time. Figure 4B shows that the extracellular erythromycin concentration of the Kam3-AcrB cells without the addition of EDHB increased from 69.67 ± 0.72 (t = 0 min) to 86.12 ± 19.63 μg/mL (t = 20 min), indicating a continuous efflux of erythromycin by the Kam3-AcrB cells. The extracellular erythromycin concentration of the Kam3-AcrB cells in the presence of EDHB decreased from 47.38 ± 2.30 (t = 0 min) to 26.85 ± 7.32 μg/mL (t = 20 min), suggesting an influx of erythromycin.

Berberine is a plant alkaloid that has EPI property against the *S. aureus* NorA, but it was able to intercalate with DNA interfering with the fluorescence of EtBr [32]. To overcome the possible interference from the putative EPIs on the fluorescence studies, high-performance LC–electrospray ionization–MS (LC-ESI-MS) [33] and MALDI-TOF [34] were reported to determine the intracellular and extracellular concentration of drugs for the detection of drug efflux. In this study, we exploited MALDI-TOF to determine that EDHB could reduce the erythromycin efflux by the transporter AcrB in *E. coli*, suggesting its potential as an EPI.

### 2.6. Molecular Docking of EDHB

**Figure 5:**
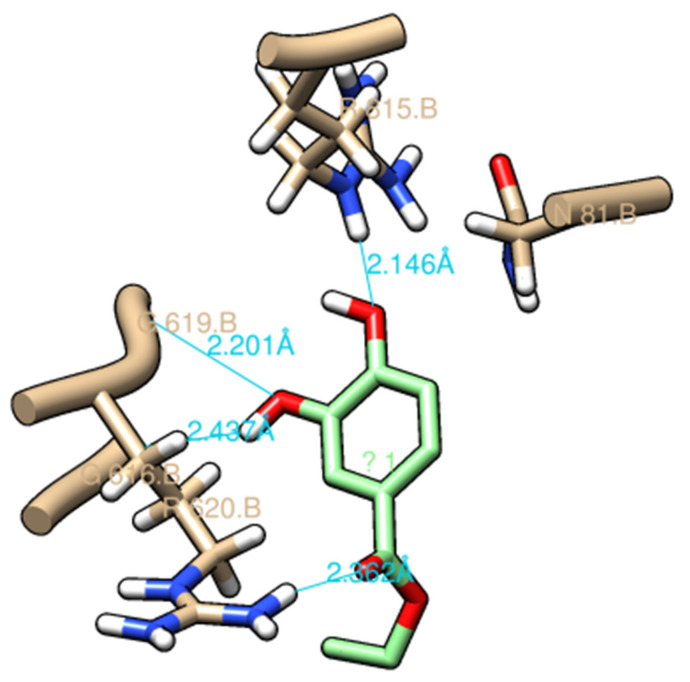
Closer view of molecular docking of EDHB to AcrB T-state monomer. Residues and hydrogen bonds (blue line) are shown within 3.5 Å from the EDHB.

Previous structural studies have proposed the drug-binding pockets, drug entrance and efflux pathways for the AcrB transporter [35]. The drug transport occurs via cooperative rotation among three monomer conformations: loose (L), tight (T), and open (O) [36]; a switch-loop (615FGFAGR620) was found to separate the PBP and the DBP. The conformational flexibility of this region is proposed to be essential for relating to substrate binding and export [37]. Based on the dye accumulation/efflux and MALDI-TOF MS data, the EDHB was shown the potency as an EPI. The blind ensemble docking of EDHB was performed using the UCSF Chimera, and the AcrB model 4DX5 was selected as the template due to its good resolution (1.9 Å) [38]. The blind docking was performed using Autodock Vina [39] by adapting a search space of size 25Å × 25Å × 25 Å in the known binding pocket region. The ensemble docking ligands were located inside the DBP of the tight state of the AcrB monomer, which is known as the putative binding site for low molecular mass substrates. As shown in Figure 5, the EDHB putatively poses within the DBP and in close interaction with the residues by H-bonds (residues G616, G619, R620, and R815) and hydrophobic contacts (residues Q89, F617, R620, and R815). In addition, the residues Q89, F617, and R620 involved in hydrogen bonding are located inside the DBP region, and residues G616 and G619, involved in hydrophobic contacts, belong to the switch loop region [40]. Vargiu and Nikaido [40] reported that the 1-(1-naphthylmethyl)-piperazine (NMP) mainly interacted with residues Q89, F136, F615, F617, and R620 when it is stacked on the side of the DBP. Moreover, many inhibitors were observed to have similar binding residues, such as F136, F178, I277, F610, V612, F615, F617, and F628 [41].

### 2.7. Effect of EDHB on the Membrane Permeability of Kam3-AcrB

Plasma membrane integrity is essential for cell function and viability; an increase in membrane permeability could lead to dissipation of the proton motive force and impairment of intracellular pH homeostasis. In this study, we investigated the effect of EDHB on bacterial cell membrane permeability.

**Figure 6:**
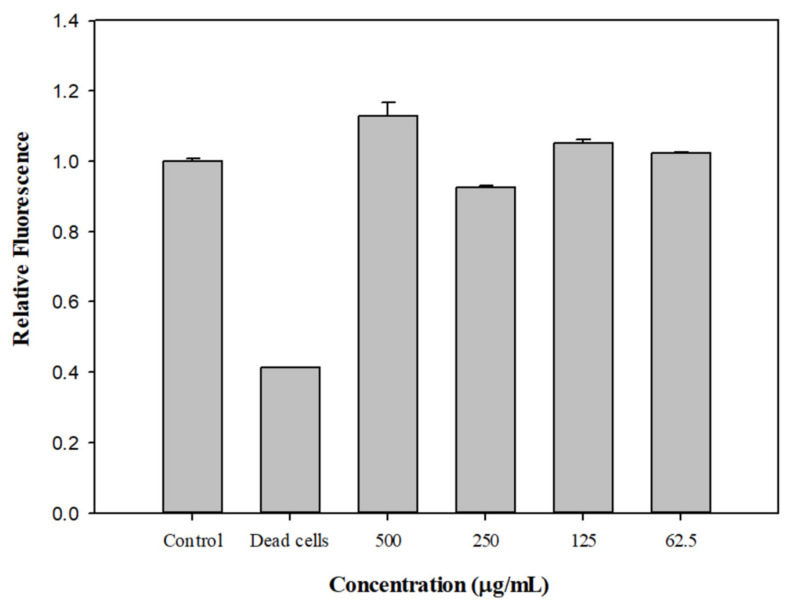
Effect of EDHB on the membrane permeabilization of *E. coli* Kam3-AcrB. Membrane permeability was determined by using fluorescence dyes SYTO9 and prodium iodide, and the fluorescence was recorded at an Ex of 470 nm and an Em of 540 nm. Data are expressed as mean ± SD (*n* = 3).

Figure 6 shows that the fluorescence of SYTO9 from the heat-inactivated dead cells was significantly reduced compared with that from the control. SYTO9 is a membrane-permeable fluorescent nucleic acid dye that is used to stain live and dead Gram-positive and Gram-negative bacteria. Membrane-impermeable propidium iodide can disrupt cell membranes to compete for SYTO9 for DNA binding, thus reducing the SYTO9 fluorescence in the dead cells. Intriguingly, the addition of EDHB at 500, 250, 125, and 62.5 μg/mL did not reduce the SYTO fluorescence, suggesting that EDHB did not increase the cell membrane permeability. CCCP has been shown to potentiate a wide variety of antibiotics against bacteria. For example, CCCP could revive the activity of tetracycline against the pathogen *Helicobacter pylori* [42] and the activities of macrolides and fluoroquinolones against *Klebsiella oxytoca* [43]. However, CCCP, a protonophore, was reported to increase membrane permeability and reduce ATP production by interfering with the transmembrane electrochemical gradient and proton motive force, making it very difficult to be applied in clinical treatment [44]. Our data indicate that EDHB did not increase the bacterial membrane permeability at the tested concentrations, which might be an advantage for its application as an EPI.

### 2.8. PAE of EDHB and Antibiotics on E. coli Kam3-AcrB

The PAE refers to the temporary suppression of bacterial growth following antibiotic treatment [45], and it can be evaluated by measuring the time of bacteria resuming growth after a short on–off exposure to an antibiotic. Supplementary Table S1 shows that the PAE for Kam3-AcrB in the presence of erythromycin was 0.30 ± 0.03. The combined use of erythromycin and EDHB significantly increased the PAE of erythromycin for Kam3-AcrB, with a PAE of 0.41 ± 0.03. The PAE for Kam3-AcrB in the presence of clarithromycin was 0.27 ± 0.03. The combined use of clarithromycin and EDHB significantly increased the PAE of clarithromycin for Kam3-AcrB to 0.37 ± 0.02. Kalia et al. [25] indicated that the plant phenolic EPI capsaicin could increase the PAE of ciprofloxacin for the Gram-positive bacterium *S. aureus* expressing efflux pump NorA by 0.5–1.1 h. For example, a combined use of ciprofloxacin (8 μg/mL) and EPI capsaicin (25 μg/mL) could extend the PAE for *S. aureus* from 1.3 h (8 μg/mL ciprofloxacin alone) to 2.4 h. However, studies regarding the use of EPIs to extend the PAE of antibiotics for Gram-negative bacteria have been very limited. Our data indicate that the use of EDHB could increase the PAE of erythromycin and clarithromycin for Kam3-AcrB by about 0.1 h, though it was relatively mild compared with the effects by other EPIs on the PAE of Gram-positive bacteria. Additionally, the overall PAE of erythromycin or erythromycin + EDHB for Kam3-AcrB was significantly smaller than the observed PAE of the antibiotics for the Gram-positive *S. aureus* [25]. Srimani et al. [45] proposed that drug detoxification via the efflux pump could explain the postantibiotic effects in bacteria following antibiotic treatment. The limited effect of EPI on the PAE for Gram-negative bacteria might be caused by the efficient efflux systems in Gram-negative bacteria, which provide faster drug detoxication.

### 2.9. Cytotoxicity Test of EDHB

**Figure 7:**
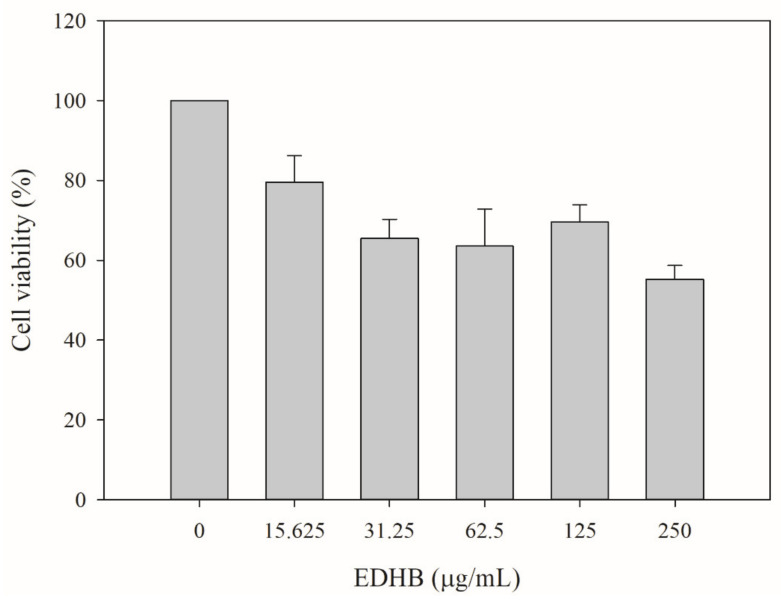
Cytotoxicity test of EDHB on human hepatic HepG2 cells. Data are expressed as mean ± SD (*n* = 3).

The possible in vitro cytotoxicity of the putative EPI EDHB to human liver cells was accomplished by monitoring the cell viability of HepG2 cells in the presence of various concentrations of EDHB. Each EDHB sample was dissolved in ethanol, while ethanol alone was used as the vehicle control (0 µg/mL of EDHB). Figure 7 shows that the cell viability for EDHB at 15.6, 31.3, 62.5, 125, and 250 µg/mL was 79.5% ± 6.6%, 65.5% ± 4.8%, 63.6% ± 9.3%, 69.5% ± 4.3%, and 55.2% ± 3.4%, respectively, as compared with that of the vehicle group. The IC_50_ of EDHB to HepG2 cells was determined to be > 250 μg/mL.

Phenothiazine chlorpromazine, currently clinically used as an antipsychotic medication in the treatment of schizophrenia and manic-depressive illness, has been shown to potentiate a wide variety of antibiotics against the bacterial strains *E. coli*, *Salmonella*, and *S. aureus* at the concentrations of 8–200 μg/mL [46]. Additionally, Machado et al. [47] showed that the IC_50_ of chlorpromazine against human monocyte-derived macrophages was 7 μg/mL (22.2 μM). Jafri et al. [48] observed that the viability of the human Hela cells was reduced by 30.1%, 50.73%, and 66.46% with the incubation of 0.050 mM, 0.1 mM, and 0.2 mM piperine, respectively, compared with untreated cells. Lu et al. [49] demonstrated that the viability of the human Hela cells gradually decreased as the DPM concentration increased, with an IC50 > 250 µg/mL. Our cytotoxicity data show that the IC_50_ of EDHB was >250 μg/mL (>1.4 mM) to human HepG2 cells, and our modulation data indicate that the phenolic compound EDHB could potentiate the antibiotics clarithromycin, erythromycin, and ciprofloxacin against *E. coli* Kam3-AcrB at a concentration of 125 μg/mL, 31.25 μg/mL, and 3.9 μg/mL, respectively, indicating that EDHB could revive the antibiotic activities at its subhalf inhibitory concentration, possibly by interfering the drug efflux of *E. coli* Kam3-AcrB.

## 3. Materials and Methods

### 3.1. Bacterial Strains, Constructs, Media, and Chemicals

*E. coli* Kam3 (DE3), which is the acrB-deleted strain, was used for genetic cloning [50], and *E. coli* Kam3 (DE3) harboring the pSYC plasmid encoding acrB (Kam3-AcrB) was obtained from Lu et al. [34] and used for drug susceptibility, modulation, drug accumulation, efflux inhibition, membrane permeability, and postantibiotic effect assays. The bacteria were grown in Luria–Bertani broth (LB broth) and Mueller–Hinton broth (MH broth) for *E. coli* cultivation and broth microdilution experiments. Erythromycin, clarithromycin, ciprofloxacin, and ethyl 3,4-dihydroxybenzoate were purchased from Sigma-Aldrich (St. Louis, Missouri, U.S.A). EDBH stock was prepared by dissolving EDHB in 95% ethanol, and it was diluted 100-fold in PBS buffer, and media for the experiments in this study.

### 3.2. IC_50_ and Modulation Tests

The IC_50_ experiments were accomplished according to Soothill et al. [51] with some modifications. The IC_50_ of the antibiotics ciprofloxacin, erythromycin, and clarithromycin against drug-resistant *E. coli* strain Kam3-AcrB was determined by using microdilution methods. The modulation assays were performed using sterile 96-well microtiter plates containing the tested antibiotics and EDHB in twofold serial concentrations. A serial dilution of the tested drugs in MH medium was performed from rows A to H in a 96-well plate, and a serial dilution of EDHB was performed from columns 1 to 12. The mixture in each well containing a drug and EDHB (20 μL) was added with 180 μL of *E. coli* Kam3-AcrB (5 Log CFU/mL), and the OD600 was determined by using a plate reader after 12 h incubation at 37°C. The modulation factors for each antibiotic, along with their EPI concentrations, were recorded. The used EDHB concentration for the modulation of ciprofloxacin, erythromycin, and clarithromycin was 3.9, 31.25, and 125 µg/mL, respectively.

### 3.3. Time-Kill Assays

The time-kill experiments were carried out according to a previous study [52], with some modifications. The time-kill study of erythromycin (250 μg/mL) alone or in the presence of EDHB (31.25 and 62.5 μg/mL) was performed in 50 mL volume conical flasks containing 20 mL *E. coli* cells (5 Log CFU/mL). The cell counts at each time point were determined by using plate counts, and each analysis was done in triplicate.

### 3.4. Drug Accumulation Assay

The H333242 and ethidium bromide (EB) accumulation assays were performed according to previous studies [53,54], with the following modifications. The *E. coli* Kam3-AcrB cells were grown to midlog phase in M–H broth and collected by centrifugation (5000× *g*, 5 min and 4 °C). The cells were resuspended twice in phosphate buffered saline containing 10 mM Na_2_HPO_4_, 1.8 mM KH_2_PO_4_, 0.5 mM MgCl_2_, 1 mM CaCl_2_, 2.7 mM KCl, and 137 mM NaCl at pH 7.4 and diluted in PBS to a final OD600 of approximate 0.5. The cell suspension (150 μL) was incubated in a 96-well plate with the filter-sterilized glucose to a final concentration of 25 mM, the H33342 (1 μM)/EB (2 μM), PAβN (20 μg/mL), and EDHB at various concentrations. The fluorescence of H33342 and EB was determined over 38 min at excitation and emission wavelengths of 360 nm and 460 nm, and 520 nm and 600 nm, respectively.

### 3.5. Drug Efflux Assay

The dye efflux assay was carried out as previously described with the following modifications [55]. The *E. coli* Kam-AcrB cells were incubated to midlog phase in M–H broth and collected by centrifugation (5000 × *g*, 5 min and 4 °C). The cells were resuspended twice in PBS buffer and diluted in PBS in a final OD600 of 0.6. The *E. coli* cells left at RT for 9 h, and incubated with H33342 or EB (3 μM), for another 30 min. The cells were resuspended twice in PBS buffer and incubated in 96-well plates with the filter-sterilized glucose (25 mM), PAβN (20 μg/mL), and various concentrations of EDHB at room temperature. The fluorescence was measured over 38 min for dye efflux measurement (Ex 360 nm and Em 460 nm for H33342; Ex 520 nm and Em 600 nm for EtBr; Ex 550 nm).

### 3.6. Monitoring Drug Efflux Using MALDI-TOF Mass Spectrometry

Monitoring drug efflux using MALDI-TOF mass spectrometry was accomplished according to Lu et al. [34] with some modifications. Various concentrations of erythromycin were dissolved in 10 mM ammonium bicarbonate buffer and mixed with matrix 2,5-dihydroxybenzoic acid for MALDI-TOF MS analysis to generate a standard curve. The *E. coli* Kam3-AcrB cells were cultivated at 37 °C to an OD600 of 0.6 to 0.8 in Mueller–Hinton broth, and the cells were collected by using centrifugation (6000× *g* for 5 min at 4 °C). The cells were resuspended twice and diluted in ammonium bicarbonate buffer (pH = 7.5) to a final OD600 of 0.6. To monitor the erythromycin efflux efficiency by transporter AcrB using MALDI-TOF, the *E. coli* Kam3-AcrB cells were added with erythromycin, EDHB, and filter-sterilized glucose, and the samples were taken from the cell mixtures at various time points for 20 min. Once collected, the sample at each time point was centrifuged (6000× *g* for 1 min) to obtain the supernatant for MS analysis. The samples were mixed with matrix 2,5-dihydroxybenzoic acid and analyzed by using MALDI-TOF MS. Data acquisition was performed automatically (random walk mode) in steps of 15 shots for a total of 3000 shots per sample. Mass spectra were analyzed by FlexAnalysis (version 3.0, Bruker Daltonics, Billerica, USA), and the ion abundancy of erythromycin from each spot was derived by the integration of signal.

### 3.7. Molecular Docking

The crystal structure of the AcrB transporter was obtained from Protein Data Bank (PDB code: 4DX5). The 4DX5 model was chosen as the template for the EDHB (PubChem CID: 77547) docking study by UCFS Chimera version 1.16. Blind ensemble docking analyses were carried out using AutoDock Vina with default parameters [39]. The docking region was chosen inside the DBP (distal binding pocket, DBP) and PBP (proximal binding pocket) of the tight state of the AcrB monomer with the search space of size 25 Å × 25 Å × 25 Å. The docking result was visualized using UCFS Chimera [56].

### 3.8. Membrane Permeability Assay

The membrane permeability assay was accomplished as previously described with some modifications [47]. *E. coli* Kam3-AcrB was cultivated in MH broth until an OD600 of approximate 1, and the bacterial cells were centrifuged (5000× *g*, 5 min and 4 °C) and resuspended in PBS to a final OD600 of 0.5–0.6. The bacterial solution was mixed with the dye mixture (SYTO 9 to propidium iodide ratio equals 1:1) in a ratio of 1: 1 and placed into a black 96–well plate in the presence of EDHB (500, 250, 125, and 62.5 μg/mL). The mixtures were incubated for 15 min at RT in the dark before the fluorescence measurement (Ex: 470 nm, Em: 540 nm).

### 3.9. Postantibiotic Effect Assay

This experiment was accomplished as previously described [57]. *E. coli* Kam3-AcrB was cultivated until midlog phase, and the bacterial cultures were divided and added with none (control), the antibiotics (2 × IC_50_ concentrations), and the antibiotics (2 × IC_50_ concentrations) + EDHB (31.25 μg/mL). The bacterial cultures were cultivated for another 2 h before inoculation into new LB broth with a thousand dilution. The cell counts (CFU/mL) were measured every hour from 0 h by using plate counts until a tenfold of the initial cell counts was reached, and the time required for the bacteria to increase by 1 log was determined.

### 3.10. Cell Toxicity Assays

The cytotoxicity of EDHB on HepG2 cells was determined as previous described [58], with some modifications. Approximately 4 × 10^4^ HepG2 cells were seeded in a 12-well culture plate and then incubated overnight. Cells were incubated with various concentrations of EDHB (0, 62.5, 125, 250, and 500 µg/mL) for 24 h before cell viability assay. Human hepatic HepG2 cells were maintained according to the instructions from Bioresource Collection and Research Center (BCRC, Hsinchu, Taiwan). All of the reagents for cell culture were obtained from Gibco/Thermo Fisher Scientific Inc. (Bethesda, MD, USA).

### 3.11. Statistical Analysis

Data were analyzed statistically by using SPSS version 12 (SPSS Inc. company, Chicago, IL, USA) and presented as mean ± standard deviation. One-way analysis of variance (ANOVA) was used to determine statistical differences between sample means, with the level of significance set at *p* < 0.05, and multiple comparisons of means were accomplished by Tukey test.

## 4. Conclusions

The use of EPI has been rapidly gaining attention as a promising approach to treat infections caused by pathogens expressing drug-resistant efflux pumps. However, one of the major drawbacks of EPI is the unacceptable level of toxicity. Therefore, EPIs of natural origin have been drawing a lot of attention. The Gram-negative bacterium *Escherichia coli* is regarded as a representative indicator of antimicrobial resistance. In this study, we evaluated the food-related phenolic compound EDHB for its EPI activity against drug-resistant *E. coli*. Our data indicate that EDHB could potentiate antibiotic activity for drug-resistant *E. coli* by interfering with the efflux pump. Additionally, EDHB did not seem to change the bacterial membrane permeability and exhibited mild human cell toxicity. In conclusion, EDHB could serve as a potential EPI for drug-resistant *E. coli*, and further in vivo experiments and pharmacokinetic studies should be taken in the future to support clinical efficacy.

## Figures and Tables

**Table 1:** Modulation assay of EDHB and the antibiotics on *E. coli* Kam3-AcrB.

Antibiotics	EDHB Concentration (µg/mL)	IC_50_ (µg/mL)			Modulation Factor	
		Alone	With EDHB	With PAβN	EDHB	PAβN
Clarithromycin	125	175	43.75	21.87	4	8
Erythromycin	31.25	125	31.25	31.25	4	4
Ciprofloxacin	3.9	0.06	0.03	-	2	-

EDHB, Ethyl 3,4-dihydroxybenzoate.

## Supplementary Materials

The following are available online at https://www.mdpi.com/article/10.3390/antibiotics11040497/s1, Table S1: Post-antibiotic effect of EDHB and antibiotics on *E. coli* Kam3-AcrB.
