# Supplementary material for: Phenolic Compound Ethyl 3,4-Dihydroxybenzoate Retards Drug Efflux and Potentiates Antibiotic Activity
Source: Antibiotics (Basel). 2022 Apr 8;11(4):497. doi: 10.3390/antibiotics11040497 (PMC9029221; doi:10.3390/antibiotics11040497)
Supplement: Supplementary file 1 [file antibiotics-11-00497-s001.zip › antibiotics-1651948-supplementary.pdf]

antibiotics

MDPI

Supplementary File

Table S1. Post-antibiotic effect of EDHB and antibiotics on *E. coli* Kam3-AcrB.

Regimen	Mean PAE (h) ± SD	
	Erythromycin	Clarithromycin
Drug	0.30 ± 0.03	0.27 ± 0.03
Drug + EDHB	0.41 ± 0.01	0.37 ± 0.02

Values are expressed as mean ± standard deviation (SD) (n = 3).
